# Ischemic stroke outcome after promoting CD4+CD25+ Treg cell migration through CCR4 overexpression in a tMCAO animal model

**DOI:** 10.1038/s41598-024-60358-2

**Published:** 2024-05-03

**Authors:** Seowoo Lee, Jiwon Kim, Je Sung You, Young-Min Hyun, Jong Youl Kim, Jong Eun Lee

**Affiliations:** 1 Department of Anatomy, Yonsei University College of Medicine, 50-1 Yonsei-ro, Seodaemun-gu, Seoul, 03722 Republic of Korea; 2 Graduate School of Medical Science, Brain Korea 21 Project, Yonsei University College of Medicine, Seoul, Republic of Korea; 3 Brain Research Institute, Yonsei University College of Medicine, Seoul, Republic of Korea; 4 Department of Emergency Medicine, Gangnam Severance Hospital, Yonsei University College of Medicine, 211 Eonju-ro, Gangnam-gu, Seoul, 06273 Republic of Korea

**Keywords:** Neuroimmunology, Stroke

## Abstract

The importance of neuroinflammation during the ischemic stroke has been extensively studied. The role of CD4+CD25+ regulatory T (Treg) cells during the recovery phase have shown infarct size reduction and functional improvement, possibly through the mitigation of inflammatory immune responses. We aimed to investigate the molecular factors involved in microglia-Treg cell communication that result in Treg trafficking. First, we observed the migration patterns of CD8+ (cytotoxic) T cells and Treg cells and then searched for chemokines released by activated microglia in an oxygen–glucose deprivation (OGD) model. The transwell migration assay showed increased migration into OGD media for both cell types, in agreement with the increase in chemokines involved in immune cell trafficking from the mouse chemokine profiling array. MSCV retrovirus was transduced to overexpress CCR4 in Treg cells. CCR4-overexpressed Treg cells were injected into the mouse transient middle cerebral artery occlusion (tMCAO) model to evaluate the therapeutic potential via the tetrazolium chloride (TTC) assay and behavioral tests. A general improvement in the prognosis of animals after tMCAO was observed. Our results suggest the increased mobility of CCR4-overexpressed Treg cells in response to microglia-derived chemokines in vitro and the therapeutic potential of Treg cells with increased mobility in cellular therapy.

## Introduction

Ischemic stroke is caused by blockage of the blood vessels leading to the brain, commonly due to thrombosis or embolism. Blockage of cerebral blood flow leads to oxidative stress and nutrient depletion, causing neuronal necrosis and the release of inflammatory factors, such as damage-associated molecular patterns, and an immune response in the brain^1,2^.

Neuroinflammation exacerbates secondary damage after a stroke, resulting in negative clinical outcomes in patients^1–7^. The ischemic penumbra is exposed to oxidative stress, excitotoxicity, and mitochondrial dysregulation, further aggravating damage. The neuroinflammatory response also leads to the release of various molecules, such as matrix metallopeptidase 9 (MMP-9), which disrupts the blood–brain barrier (BBB)^1,4^. Breakdown of the BBB augments secondary damage by allowing pro-inflammatory immune cell infiltration into the ischemic brain. Within a few hours, peripheral responders, such as neutrophils, monocytes, and CD8+ (cytotoxic) T cells, enter the brain^3,6^.

Immediate responders to ischemic stroke are brain-resident microglia, which undergo functional and morphological changes into classical M1 or alternative M2 phenotype^8,9^. The M1 phenotype exerts a pro-inflammatory response, releasing reactive oxygen species and inflammatory cytokines like interleukin-6 (IL-6) and tumor necrosis factor-α (TNF-α). Conversely, the protective M2 phenotype releases transforming growth factor-β (TGF-β) and interleukin-10 (IL-10) and activates the debris clearance process by phagocytosis^8,10^. Additionally, chemoattractants secreted by activated microglia further induce the migration of immune cells, such as monocytes and pro-inflammatory T cells, into the ischemic brain^11^. Many previous studies have focused on manipulating M1 microglia to reduce neuroinflammation^8,9,12^.

Ischemic stroke is the most prevalent type thereof, and the AHA Statistical Update has reported a steady overall increase in the incidence of ischemic stroke and related deaths^6^. Although stroke is a major health threat, recombinant plasminogen activator (tPA) is the only current Food and Drug Administration-approved treatment. Temporal and geographical limitations of tPA necessitate the identification of other effective treatments. In the search for novel treatments, studies based on cell therapy, especially using CD4+CD25+ regulatory T (Treg) cells, are ongoing.

Studying the role of Treg cells in immune homeostasis in autoimmune diseases and cancer is the main focus of Treg cell research^13^. Most studies have focused on the ability of cytotoxic T cells to suppress antitumor immune responses. More recently, research has shown repair potential in various contexts, such as cardiovascular injury, lung infections, and central nervous system (CNS) repair after spinal cord injury and stroke^14–16^.

Concerning the influx of pro-inflammatory immune cells upon the breakdown of the BBB, Treg cells, known for their anti-inflammatory response, also migrate to the infarct site^5,7^. Treg cells and their anti-inflammatory functions have neuroprotective effects in the ischemic brain with respect to infarct size reduction and neuron recovery^17^. Treg cells also express inhibitory molecules such as CTLA-4 and programmed cell death ligand 1 (PD-L1), suppressing pro-inflammatory immune cells. Furthermore, Treg cells interacting with the activated microglia lead to the alteration of M1 microglia to M2 by releasing IL-10 and TGF-β^18^. Successful suppression of pro-inflammatory IL-17 + γδ T cell and MMP-9 secretion has been seen in concert with the increase and accumulation of Treg cells in the circulation and the ischemic penumbra^19,20^. Treg cells are known to accumulate in the ischemic penumbra region within 24 h but are more prominent during the recovery phase, generally after a week and up to 6 months post-stroke^21^. It has been proposed that Treg cells suppress the growth of the infarct area and neuroinflammation, ultimately preventing the aggravation of neuronal damage and promoting tissue repair^3,20^.

Concerning previous studies on the role of microglia in recruiting peripheral immune cells into the ischemic brain, the molecular mechanism concerning Treg cell trafficking into the ischemic brain is still to be elucidated. Further clarification is required regarding the therapeutic role of Treg cells in the acute phase of ischemic stroke. We, therefore, aimed to identify the contributors to Treg cell migration into the ischemic brain and their effects on infarct size and neurological and motor functions in tMCAO animal models.

## Materials and methods

### Animals

C57Bl/6 mice (male, 8–10 weeks old) were purchased from Orient Bio, Inc. (Gyeonggi, South Korea) and were housed in a pathogen-free (SPF) environment at the Avison Biomedical Research Center, Yonsei University (Seoul, South Korea). All experiments were performed following the guidelines of the Institutional Animal Care and Use Committee of Yonsei University Health System (IACUC number: 2020-0262) and the National Institutes of Health. All animal experiments were planned, performed, and analyzed according to the ARRIVE 2.0 Essential 10 guidelines^22^.

### Microglia isolation, culture, and oxygen–glucose deprivation (OGD)

All procedures were performed according to the protocol provided by Miltenyi Biotec (Auburn, California). The brain was isolated from a C57Bl/6 mouse, and tissues were homogenized using the Adult Brain Dissociation Kit, mouse, and rat (Miltenyi Biotech) and the Miltenyi GentleMACS system. The homogenized brain suspension was collected to isolate microglia using CD11b (Microglia) MicroBeads (Miltenyi Biotech). Microglia were seeded at a density of 1.0 × 10^6^/mL in complete RPMI media (10% FBS, 1% penicillin–streptomycin, and 0.5 mM HEPES). The complete RPMI media from the primary microglia culture was removed, collected (Control media), and replaced with an RPMI-only media. Primary microglia were exposed to OGD for 40 min (OGD media) using the Thermo Forma Anaerobic System Model 1025 (Thermo Fisher Scientific, Inc., Waltham, Massachusetts). After exposing the microglia to mild OGD conditions, the media was replaced with a complete RPMI media (OGDR media). Microglial cultures were recovered under standard culture conditions for 24 h.

### Primary T cell isolation, culture, and activation

All procedures were performed according to the protocol provided by Miltenyi Biotec. The spleen was dissociated using a Spleen Dissociation Kit (Miltenyi Biotech) and homogenized using the Miltenyi GentleMACS system. Splenocytes were further processed using the mouse CD8a+ T Cell Isolation Kit and the CD4+CD25+ Regulatory T Cell Isolation Kit (Miltenyi Biotech). Isolated cells were cultured at a density of 1.5 × 10^6^/mL in complete RPMI media with recombinant IL-2 (Sigma-Aldrich, St. Louis, Missouri) at 2500 U/mL. Cells were seeded at a density of 1.5 × 10^6^/mL in a 24-well flat-bottom plate. Isolated cells were activated overnight with Gibco Dynabeads Mouse T-Activator CD3/CD28 for T Cell Expansion and Activation (Thermo Fisher Scientific, Inc.). Cytotoxic T cells were activated at a T cell:bead ratio of 1:1 and Treg cells at a Treg:bead ratio of 1:2.

### Transwell migration assay and Treg cell antagonist treatment

We placed 600 μL of collected microglia culture media into each well of the 24-well plate. Then, 24-well SPLInsert™ Hanging (SPL, Gyeonggi-Do, Korea) with 200 μL of cell suspension containing 9.0 × 10^5^ of primary CD8+ T cells or Treg cells were inserted. The cells were cultured under standard culture conditions for 48 h. The number of migrated cells was counted using 33342 Hoechst dye (Thermo Fisher Scientific, Inc.). After removing the transwell inserts before imaging, the plates were briefly centrifuged. The CCR4 inhibitor (sc-221406) was obtained from Santa Cruz Biotechnology, Inc. (Dallas, Texas), and the CXCR4 inhibitor (WZ811) was obtained from MedChemExpress (Monmouth Junction, New Jersey). Treg cells were incubated with each inhibitor for 2 h under standard culture conditions, and 0.02 mg/mL DMSO was added to the antagonist-treated Treg cells as a negative control.

### Mouse chemokine profiling array and western blot analysis

Each media collected from the microglial OGD experiment was analyzed using the Proteome Profiler Mouse Chemokine Array Kit (R&D Systems Inc., Minneapolis, Minnesota) following the protocols provided by the manufacturer. The intensity of each dot was measured using ImageJ software. One milliliter of the collected microglia culture media was incubated with the provided cellulose membrane, which was then incubated in Chemi Reagent Mix (Thermo Scientific, Inc.) and visualized in LAS 4000 mini (Fujifilm, Tokyo, Japan). Western blotting was performed as previously described^23^, whereby 30 µg samples were separated in 4–20% Mini-PROTEAN® TGX™ Precast Protein Gels (Bio-Rad, Hercules, California). Detection of each receptor was performed using the following antibodies: rabbit anti-CXCR4 UMB2 (1:100, Abcam, UK, Cambridge, UK), mouse anti-CD4 9H5A8 (1:100, Invitrogen, Waltham, Massachusetts), goat anti-CCR4 (1:100, Abcam, UK), goat anti-CCR10 (1:100, Invitrogen), and β-actin (1:1000, Abcam, UK). Membranes were washed and incubated with secondary antibodies diluted in a 3% BSA solution for 2 h at room temperature (RT) on a shaker. Each antibody was detected using secondary antibodies, such as goat anti-mouse IgG HRP (1:500, Thermo Fisher Scientific, Inc.), goat anti-rabbit IgG HRP (1:500, Thermo Fisher Scientific, Inc.), and goat anti-rat IgG H&L HRP (1:500, Abcam, UK). The membranes were visualized using Pierce™ ECL Western Blotting Substrate (Thermo Fisher Scientific, Inc.). Images were captured using the chemiluminescent image analyzer LAS 4000 mini (Fujifilm, Japan), and grayscale values were measured using ImageJ. The membranes were trimmed before antibody incubation for all samples.

### Immunocytochemistry (ICC) and image analysis

The cells were fixed in 4% paraformaldehyde (PFA) and blocked with a 3% BSA solution for 1 h. Extracellular staining was performed overnight at 4 °C, followed by permeabilization with 0.1% Tween 20 for 15 min. Intracellular staining of FoxP3 was performed for 2 h on ice. 1 × DAPI (Invitrogen) diluted in 1 × PBS solution was used for counterstaining for 5 min at RT. The slides were washed, mounted with a mounting solution, and covered with a coverslip. Confocal microscopy images were obtained using a Zeiss LSM 710 microscope (Carl Zeiss, Oberkochen, Baden-Württemberg), and were processed using the ZEN 2009 Light Edition program (Carl Zeiss). Corrected total cell fluorescence (CTCF) was measured using ImageJ.

Labeling of each receptor was performed with the following primary antibodies: rabbit anti-CD8α (1:100, Abcam, UK), rabbit anti-Foxp3 EPR22102-37 (1:100, Abcam, UK), rabbit CXCR4 UMB2 (1:100, Abcam, UK), mouse CD4 9H5A8 (1:500, Invitrogen), goat anti-CCR4 (1:100, Abcam, UK), and goat anti-CCR10 (1:100, Invitrogen). This was followed by labeling each with fluorescent-labeled secondary antibodies such as goat anti-Rat IgG-Rhodamine (1:1,000, Invitrogen), donkey anti-rabbit IgG-Alexa Fluor™ 594 (1:1,000, Invitrogen), goat anti-mouse IgG-FITC (1:100, Sigma-Aldrich), donkey anti-goat IgG-FITC (1:100, Santa Cruz Biotechnology, Inc.), and donkey anti-rabbit IgG-FITC (1:1,000, Abcam, UK).

CTCF was measured using ImageJ. Each cell line was selected, and the fluorescence intensity was measured for each channel. The intensity values were normalized against DAPI intensity and then normalized against FoxP3 or CD8α intensity. The area-integrated fluorescence intensity was measured, and the CTCF was calculated using the following formula:

$$ CTCF = Integrated\;fluorescence\;intensity - \left( {Area\;of\;the\;cell \times Background\;mean\;fluorescence} \right) $$

### MSCV retrovirus production and transduction

The pMSCV Exp-mCcr4 NM_009916.2 (ns): P2A: EGFP (Vector ID: VB230314-1872tcs) vector was designed and created using VectorBuilder, Inc. (Guangzhou, China). The vector was then packaged into a recombinant MSCV retrovirus using VectorBuilder, Inc. (Guangzhou, China).

The general procedure for Treg cells was transduced as previously described^24^. Transduction media was prepared with RPMI media with 100 mM HEPES and 10 μg/mL polybrene. We carefully pipetted 100 μL of the virus stock into each well containing 1 mL of the transduction media. The culture was spinoculated at 1,200 g for 90 min at 32 °C. Cells were incubated under standard culture conditions for 4 h, followed by a media change. Treg cells were sorted on days 5 or 6 using a BD Aria III (Becton Dickinson Company, Franklin Lakes, New Jersey).

### Flow cytometry (FACS)

We washed and fixed 1 × 10^6^ untreated Treg cells or transduced Treg cells in a cold 2% PFA solution. The cells were blocked in a 2% BSA solution for 30 min on ice. The supernatant was discarded, and extracellular CCR4 staining was performed for 30 min at 4 °C with hamster anti-mouse CD194 (CCR4)-PE 2G12 (1:100, BioLegend, San Diego, California). CD8 was also stained for 30 min at 4℃ with anti-mouse CD8-PECy^7^ (1:100, BD company, New jersey). Intracellular staining was performed by permeabilizing the cells with a 0.7% Tween-20 solution for 15 min on ice. Then, Foxp3 was stained using anti- mouse FoxP3-PE (1:100, BD company), rat anti-mouse FoxP3-Alexa Fluor 700 MF-14 (1:100, BioLegend), anti-mouse FoxP3-APC (1:100, BD company), anti-mouse Ig, k/Negative control compensation beads (Neg. Con), and APC-Positive control antibody with compensation beads (Pos. con) in the dark for 30 min at 4 °C. Flow cytometry analysis was done using the BD FACSymphony™ A5 Cell Analyzer (Becton Dickinson Company). The collected data were further processed using FlowJo™ v10.8 software (Becton Dickinson Company).

### Transient middle cerebral artery occlusion (tMCAO)

The tMCAO surgery was performed using the method described by Koizumi et al.^25^. Mice were deeply anesthetized with 30 mg/kg Zoletil (Virbac, Carros, France) mixed with 10 mg/kg Rompun (Bayer, Leverkusen, Germany) via intraperitoneal injection. Briefly, the common carotid artery (CCA) and external carotid artery were exposed, and knots were made. A small incision was made near the first CCA knot, and a 6-0 fine MCAO suture L56 (6-0) (Doccol, Sharon, Massachusetts) was inserted into the middle cerebral artery. Occlusion was performed for 20 min, while the animals were kept under a heating lamp. In the sham group, all steps were performed until the insertion of the MCAO suture. In the experimental groups, after the removal of the MCAO suture, the animals were anesthetized for another 20 min under a heat lamp. Following surgery, mice were administered analgesics (Meloxicam, 5 mg/kg, once daily) and antibiotics (Gentamicin, 5 mg/kg, twice daily) via subcutaneous injection to mitigate pain and prevent infection. We prepared 1.0 × 10^6^ untreated Treg cells or MSCV-CCR4 transduced Treg cells in 40 uL saline per animal in an insulin syringe and injected them through the CCA. Since all in vivo experiments were analyzed 24 h after the tMCAO surgery.

### Behavior testing and the 2,3,5-triphenyltetrazolium chloride (TTC) assay

Behavioral testing was performed 24 h post-tMCAO. The Bederson score was used as indicated by De Meyer et al.^26^. The grading criteria were as follows: 0, no defects; 1, forelimb flexion when untouched; 2, reduced resistance to lateral push; 3, circling behavior; 4, longitudinal spinning; and 5, no movement. The forelimb grip strength test was performed as described by Takeshita et al.^27^. When the forepaws were attached to the bar, the measurement device was torn. The tail was gently pulled horizontally until the mouse could move. The number displayed on the screen was recorded in kilograms. The elevated body swing test (EBST) was conducted as described by Borlongan et al.^28^. The mice were then placed in a new cage for 2 min for habituation. The mouse was held up approximately 10 cm vertically by its tail, and the side to which it swung was recorded.

The mice were transcardially perfused with 30 mL of ice-cold saline. The brain was isolated and sliced into 2 mm sections. They were incubated in a pre-warmed 2% TTC solution in the dark for 30 min at 36 °C. Brain slices were photographed, and the infarct area was measured using ImageJ.

### Statistical analysis

All statistical analyses were done using Prism software (GraphPad Software, La Jolla, California) and were considered statistically significant at *p* < 0.05. Two-way ANOVA was used to analyze the significance between the groups, whereas the simple comparison between the means of each group in an experiment was done using the One-way ANOVA or unpaired t test. All data are presented as mean ± SD.

### Ethical approval and consent to participate

The animal study was reviewed and approved by The Institutional Animal Care and Use Committee of Yonsei University Health System and according to the National Institutes of Health guidelines.

## Results

### Cytotoxic T cells and Treg cells showed increased migration in response to inflammatory microglia-derived chemokines

**Figure 1:**
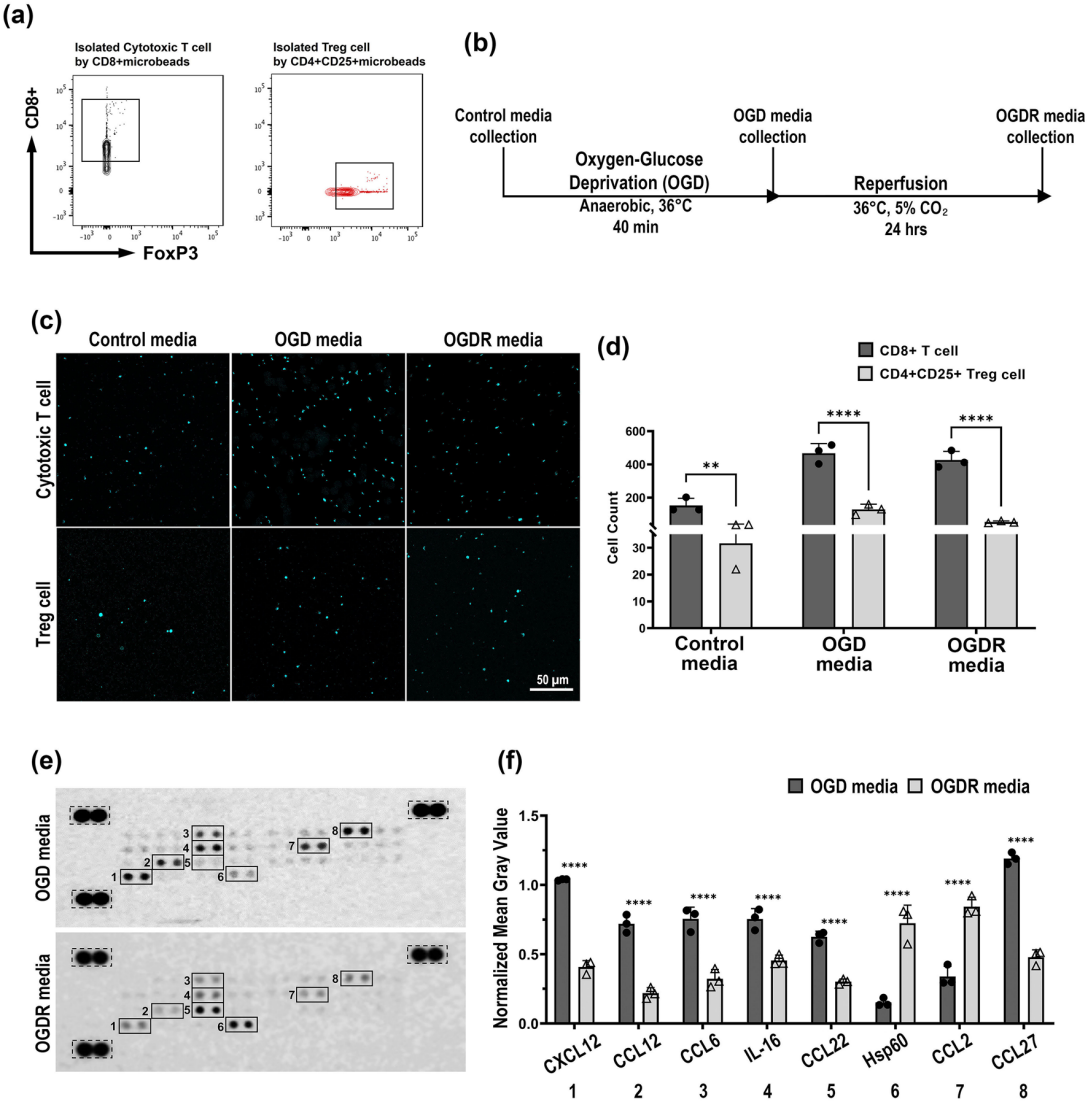
More cytotoxic T cells and Treg cells migrated into the OGD media compared to the OGDR media in response to chemokines secreted by activated microglia during OGD. (**a**) Graphical schedule regarding the microglia media collection. (**b**) FACS analysis showed CD8+ T cell and FoxP3 Treg cell isolation. (**c**) Transwell migration assay confocal imaging results after 48 h into each of the indicated primary microglia media. (**d**) The cell counts data of cytotoxic T cells and Treg cells. (**e**) Murine chemokine profiling array chemiluminescence data shown for OGD and OGDR media. All gray values for each dot blot in each media were measured relative to the reference dot blot (dotted boxes). The numbers correspond to the numbers in Table 1. (**f**) Normalized mean gray intensity value for the dot blot incubated in OGD or OGDR media. Asterisks denote the significance of data obtained by performing the Two-way ANOVA and Sidak’s multiple comparison (**p ≤ 0.01, ****p < 0.0001, all n = 3).

To isolate cytotoxic T cell and T reg cell, we used each CD8+ and CD4+CD25+ microbeads, and confirmed FACS analysis (Fig. 1a). The migration patterns of cytotoxic T cells and Treg cells were confirmed using a transwell migration assay. Microglial culture media were collected during OGD experiment (Fig. 1b). Confocal images show increased cytotoxic T cell and Treg cell migration into the OGD media (Fig. 1c). Compared to the control media, cytotoxic T cells showed a 3.1-fold increase (*p* < 0.001), whereas Treg cells showed a 4.1-fold increase (*p* < 0.01) in migration into the OGD media (Fig. 1d). Cytotoxic T cells (9.6%) and Treg cells (57.5%) that migrated into OGD + reperfusion (OGDR) media decreased compared to those that migrated into OGD media. Together, these results indicate increased migration of cytotoxic T cells and Treg cells into media where microglia are activated in response to OGD exposure.

Next, the chemokines secreted by the activated microglia were profiled using the collected microglial culture media with a mouse chemokine profiling array. The relative mean gray values dramatically increased for CXCL12, CCL12, CCL2, CCL6, IL-16, and CCL27 in OGD media (Fig. 1e,f), where CXCL12 and CCL27 showed the highest expression levels. In contrast, the OGDR media showed high levels of CCL22 and Hsp60. Comparing the differences between OGD and OGDR media, the expression levels of CXCL12 and CCL12 were 3.5-fold higher in OGD media. CCL2 levels increased 2.3-fold, whereas CCL6 levels differed 1.7-fold. IL-16 expression was approximately 2.1-fold higher in the OGD media, whereas CCL27 levels differed by 2.5-fold. In contrast, CCL22 and Hsp60 levels were 4.8- and 2.5-fold higher, respectively, in OGDR media. The activation of microglia in response to ischemic stroke result in the release of chemokines related to the inflammatory immune response.

The receptors interacting with these chemokines were investigated (Table 1). A general trend of an increase in pro-inflammatory chemokines was observed in the OGD media. Instead, the OGDR media showed an increase in chemokines related to Treg cell function. These results narrowed down the possible target receptors for this study concerning Treg cell migration into the ischemic brain in response to microglial-derived chemokines.

**Table 1:** Chemokines observed in murine chemokine arrays in conditioned primary microglia culture media and interactions with known receptors. The numbers on the left (#) represent the chemokines shown in Fig. 1d.

#	Chemokine	Receptor	Roles	References
1	CXCL12	CXCR4	T cell, B cell, monocyte, and Treg cell recruitment	^28^
2	CCL12	CCR2	Activation of microglia and complement activation in the brain	^29^
7	CCL2			
3	CCL6	CCR6	Migration of macrophage, neutrophil, astrocyte, and microglia	^30^
4	IL-16	CD4	Skin inflammation through over-sensitization, CD4+ T cell expansion CD4+ T cell differentiation into inflammatory Th17 cells	^31,32^
5	CCL22	CCR4	Suppression of T cell response through Treg cell activity	^33–35^
6	Hsp60	TLR9/TLR2	Anti-inflammatory response through inhibiting T cell migration indirectly	^36–38^
8	CCL27	CCR10	Lymphocyte recruitment, inflammation, and allergic reactions in skin Inflammatory immune response in the brain	^39^

### CCR4, highly expressed in Treg cells, may be more intimately involved in Treg cell migration

**Figure 2:**
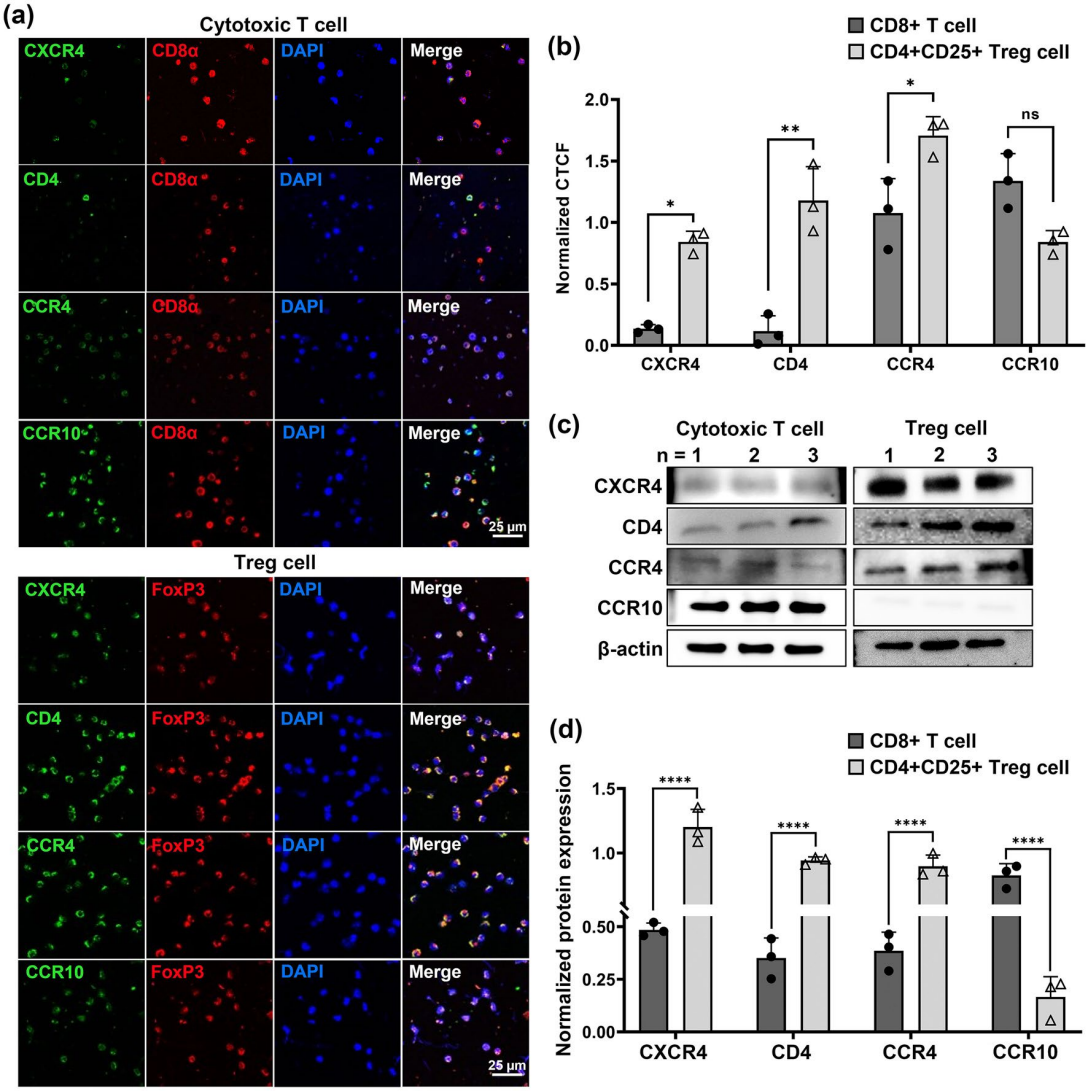
Confirmation of the selected receptors in cytotoxic T cells and Treg cells through immunocytochemistry (ICC) and western blot. (**a**) ICC confocal imaging results showing the expression of the screened receptors: CXCR4, CD4, CCR4, and CCR10 on cytotoxic T cells and Treg cells, as well as CD8α or FoxP3, respectively. (**b**) The corrected total cell fluorescence (CTCF) was measured with ImageJ and normalized against either CD8α or FoxP3, respectively. (**c**) Western blot results of the receptors listed above, as well as β-actin as a reference in cytotoxic T cells and Treg cells. Each receptor from each cell type was ran in different gels and transferred onto separate membrane (full length gels can be viewed in the Supplementary Fig. S2). (**d**) The above western blot band intensity for each receptor was measured using ImageJ and normalized against the β-actin bands in cytotoxic T cells and Treg cells. Asterisks denote the significance of data from p-values obtained by performing the Two-way ANOVA and Sidak’s multiple comparison (*p ≤ 0.05, **p ≤ 0.01, ****p < 0.0001, all n = 3).

The expression levels of the receptors investigated above were observed in cytotoxic T cells and Treg cells by ICC and western blotting. The cytotoxic T cells and Treg cells were fluorescently labeled for CD8α and FoxP3, respectively, as well as CXCR4, CD4, CCR4, and CCR10 (Fig. 2a,b). Expression levels were quantified by measuring CTCF and in western blot (Fig. 2c,d).

Treg cells showed higher CXCR4 expression than cytotoxic T cells in CTCF measurements (16.1%) and western blot (40.4%). Similar to CXCR4, CD4 (9.8%, in CTCF, 37.3%, for western blot) and CCR4 (63.0%, in CTCF, 43.0%, for western blot) expression levels were higher in Treg cells. CCR10, on the other hand, showed higher expression in cytotoxic T cells in CTCF measurements (62.9%) and in western blot (20.1%). In summary, CXCR4, CD4, and CCR4 expressions were higher in Treg cells, whereas CCR10 expression was higher in cytotoxic T cells.

The two receptors were treated with their respective antagonists to block their activity and to observe their involvement in Treg cell migration (See supplementary Fig. S1 online). Blockage of CCR4 resulted in a significant reduction in migration into OGD media compared to CXCR4 inhibition (20.2%). Hence, the target receptor used in this study was CCR4.

### Overexpression of CCR4 on Treg cells resulted in increased migration in the OGD condition

**Figure 3:**
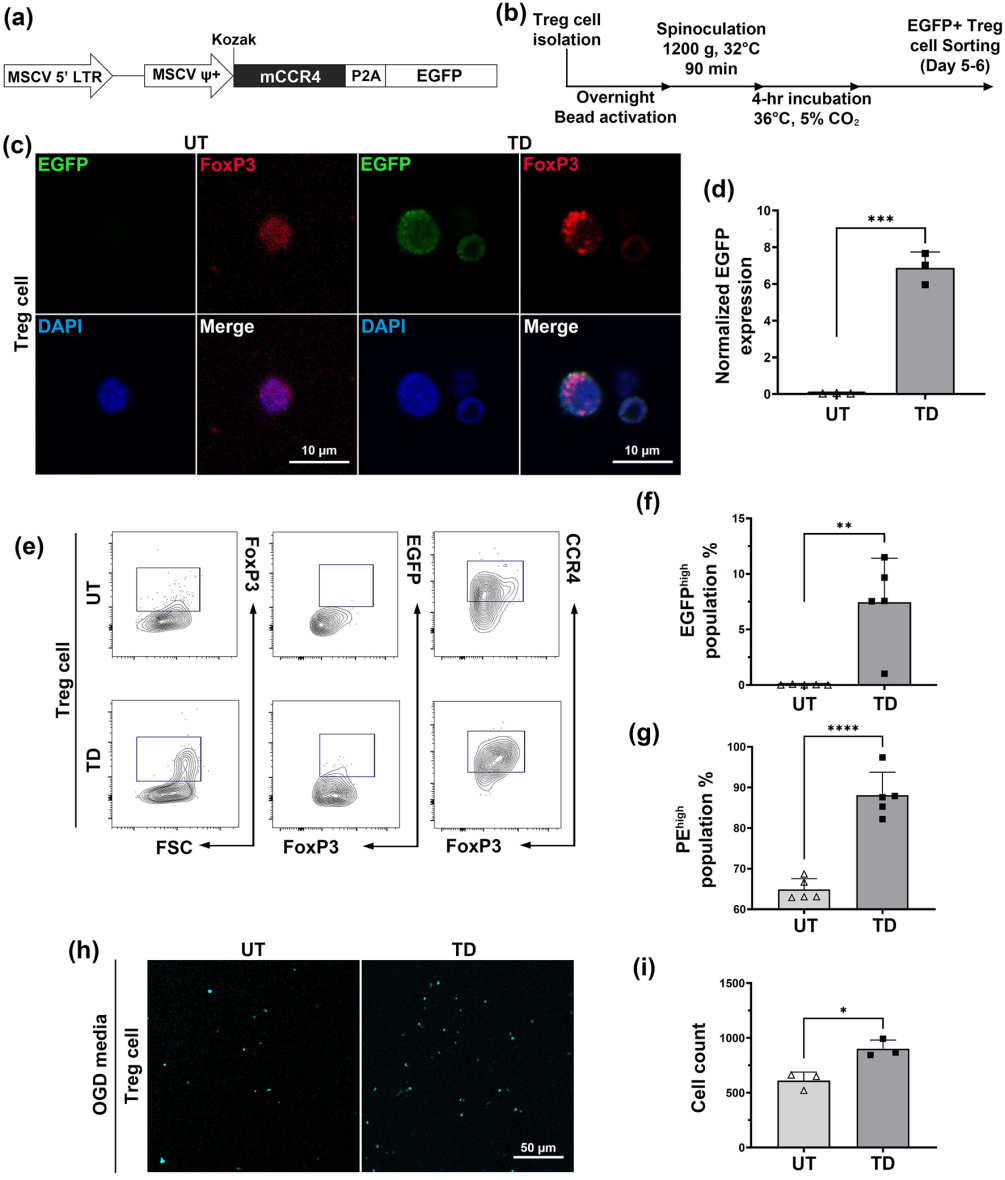
The Treg cell MSCV-CCR4 retrovirus transduction showed an increase in CCR4 expression, further increasing the migration into the OGD media. (**a**) The MSCV retrovirus vector construct was designed via VectorBuilder. The mCcr4 sequence was linked with the EGFP reporter gene with a P2A linker, where the expression was controlled by the MSCV ψ+ promoter. (**b**) Transduction was performed following this schedule. (**c**) ICC imaging of the UT and TD Treg cells. (**d**) CTCF was measured with the ImageJ program and normalized against the FoxP3 fluorescence measurements. (**e**) The detailed quantification of changes in CCR4 expression was observed using FACS. (**f**, **g**) Graph of the FoxP3+ Treg cell population expressing EGFP and CCR4 highly. (**h**) Confocal images of Treg cells that migrated into the chamber containing OGD media. (**i**) The number of Treg cells that migrated into the OGD media after 48 h. Asterisks denote the significance of the p-values obtained by performing the unpaired t-test (*p ≤ 0.05, **p ≤ 0.01, ***p ≤ 0.001, ****p < 0.0001, all n = 3–5).

MSCV retrovirus transduction was conducted to increase the migration rate of Treg cells by overexpressing CCR4. The MSCV vector contains a mouse CCR4 sequence linked with EGFP by a P2A linker (Fig. 3a). Figure 3b shows the graphical schedule for this experiment. The transduction efficiency was qualitatively and quantitatively confirmed using ICC (Fig. 3c,d) and flow cytometry (Fig. 3e–g).

Comparing the untreated Treg cells (UT) with the MSCV-CCR4-transduced Treg cells (TD), the ICC results showed a 6.8-fold increase in EGFP expression (Fig. 3d). The TD group showed a higher percentage of the EGFP population, consistent with the ICC results (7.4%) (Fig. 3e,f). Among the FoxP3 + population, CCR4-PE expression in TD was higher than that in the UT group (23.2%), indicating a mild increase in the expression levels of CCR4 in TD group (Fig. 3e,g). These results showed successful increase of CCR4 expression in Treg cells.

After confirming the successful transduction of Treg cells with the MSCV-CCR4 retrovirus, a transwell migration assay was conducted to determine the changes in the migration pattern of CCR4-overexpressed Treg cells (Fig. 3h). A 1.5-fold increase in TD migration into the OGD media was observed compared to UT (Fig. 3i). This result suggests that the overexpression of CCR4 in Treg cells increases their migration ability in response to microglia-derived CCL22 during OGD.

### CCR4-overexpressed Treg cells may exert therapeutic effects

**Figure 4:**
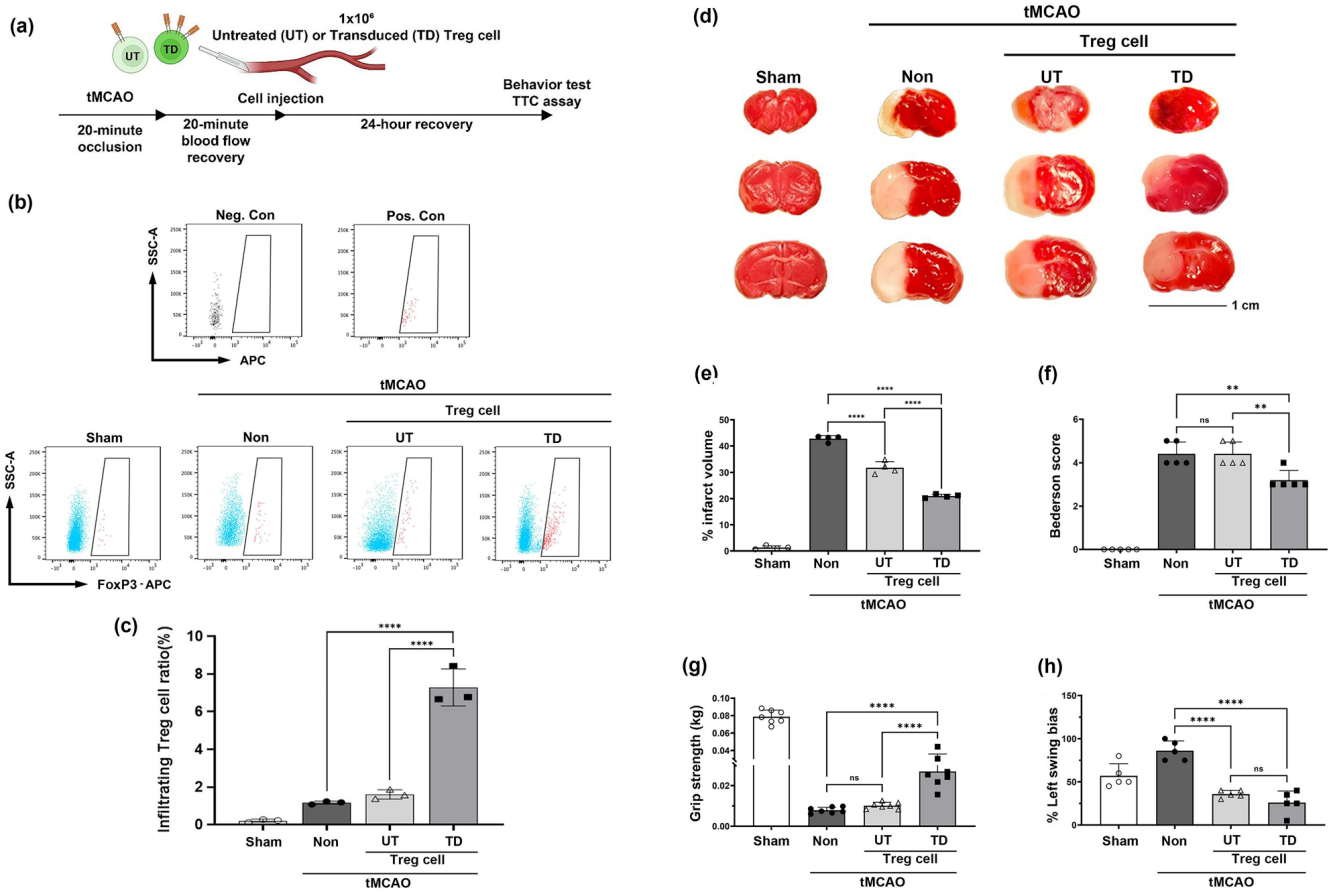
TD Treg cell injection group showed further decrease in infarct size and notable improvement in tMCAO animal model. (**a**) All experiments followed the graphical schedule shown here. (**b**, **c**) FACS analysis of Foxp3 positive cells migrated in ischemic penumbra area 24 h after tMCAO (negative control (Neg. Con), positive control (Pos. Con), ****p < 0.0001, n = 3). (**d**) TTC assay results for the indicated groups. (**e**) The infarct size was measured using ImageJ. (**f**) The Bederson score was measured to observe the overall deficits in neurological functions of animals 24 h after tMCAO. (**g**, **h**) Motor function of the animals post-tMCAO modeling was done using the forelimb grip strength test and the elevated body swing test (EBST). Asterisks denote the significance of data from p-values obtained by performing the One-way ANOVA and Sidak’s multiple comparison (**p ≤ 0.01, ****p < 0.0001, n = 4–7).

tMCAO was performed to confirm the potential therapeutic effects of Treg cells during the acute phase of ischemic stroke. Infarct size was measured using the TTC assay, and functional improvements were observed via behavioral testing following a graphical schedule (Fig. 4a). To confirm the migration of injected TD Treg cells 24 h after tMCAO, we measured FACS analysis of the FoxP3 positive cell population in the ischemic penumbra area (Fig. 4b). FoxP3 positive cells significantly increased in TD group by ~ 4.8-fold compared with the UT and non group after tMCAO (Fig. 4c). The TTC assay indicated a dramatic decrease in infarct size in UT compared to the non (25.7%) after tMCAO (Fig. 4d,e). The TD injection group showed a further reduction in infarct size compared with the non (51.0%) after tMCAO. The UT and TD group comparisons showed a 34.0% further infarct size reduction in TD group than in UT group after tMCAO (Fig. 4e).

The animals were then tested for functional improvement using the Bederson score, forelimb grip strength test, and EBST. The Bederson score assigned to each animal depended on its behavior 24 h post-tMCAO. The UT and TD groups showed a slight decrease in the severity of neurological deficits compared to the tMCAO control, the score value differences being 0.25 (ns) and 1.25, respectively (Fig. 4f). The forelimb grip strength test showed a 2.7-fold increase in the TD group, whereas the UT group showed a 1.3-fold increase compared with the tMCAO control (Fig. 4g). Finally, the EBST showed a dramatic decrease in the percentage of left swing bias in the UT and TD groups (Fig. 4h). The UT group showed a 50.2% reduction, whereas the TD group showed a 60% reduction in bias compared with the tMCAO control group. Together, these results showed a notable decrease in infarct size when TD Treg cells were injected into the animal after tMCAO surgery, as well as some improvement in neurological and motor function post-tMCAO.

## Discussion

The heterogeneity of results regarding the effects of Treg cells during the acute phase of ischemic stroke does not explain the therapeutic potential of Treg cells. This study investigated the specific molecular factors involved in Treg cell migration upon microglial activation in the brain under ischemic stress and the effects of Treg cells after tMCAO in the context of neurological and motor functions. Transwell migration assay results showed increased migration into OGD media for cytotoxic T cells and Treg cells. The murine chemokine profiling array showed increased CCL27, CCL2, and CXCL12 expression in the OGD media, whereas the OGDR media showed higher expression of Hsp60 and CCL22. ICC and western blot results showed that the receptors known to interact with the listed chemokines, blocking the CCR4 receptor, resulted in a notable decrease in Treg cell migration into the OGD media. These results suggest that microglia-derived CCL22 and CCR4 in Treg cells are key factors in Treg cell trafficking. Treg cell migration into OGD media increased upon the overexpression of CCR4 using the MSCV-CCR4 retrovirus. Injection of TD Treg cells after tMCAO resulted in further infarct size reduction and mild functional improvement. These results indicate that increased expression of CCR4 and Treg cell migration into the infarct site in response to chemokines secreted by activated microglia has noteworthy effects on reducing the severity of ischemic stroke.

Although there have been many studies on the role of Treg cells in the recovery phase of ischemic stroke^21^, conclusive evidence on the neuroprotective effects of Treg cells is still lacking. Previous studies have shown the accumulation of Treg cells in the bloodstream and ischemic penumbra. The accumulation of Treg cells around the brain infarct region and cerebral vasculature suggests that Treg cells react and migrate in response to microglia-derived factors during ischemia^5^. Treg cells are known to reduce neuroinflammation through the expression of PD-L1 to inhibit the activity of MMP-9 released by neutrophils and the activity of cytotoxic astrocytes via the amphiregulin (AREG)/epidermal growth factor receptor axis^17^. Furthermore, Treg cells provide neuroprotection indirectly through IL-10 and TGF-β secretion, shifting microglia into the M2 phenotype that promotes brain repair^18^. Recent studies have shown that Treg cell injection enhanced white matter integrity and repair and resulted in better functional recovery when Treg cell proliferation was promoted in animal models^29,30^.

The chemokine profiles in OGD media showed an increase in chemokines involved in various physiological pathways, such as nociception in pathological conditions, causing adverse effects in cancer patients^29,31^. CCL27 and CXCL12 levels showed the largest increases. CCL27 interacts with CCR10 and is the most studied in skin lymphocyte recruitment, inflammation, and allergic reactions in the skin; however, alternatively spliced CCL27 is also found in brain tissue^32^. Alternatively, spliced CCL27 is involved in neuroimmune responses, suggesting a pro-inflammatory role in the brain. In addition, the CXCL12-CXCR4 interaction is responsible for recruiting T cells, B cells, and monocytes and also induces anti-inflammatory Treg cell recruitment^33^.

Chemokines, such as CCL12, CCL2, CCL6, and IL-16, were increased in the OGD media. CCL12 and CCL2 interact with CCR2 and contribute to the expression of Iba1, thereby activating the brain complement system via C1qa^34^. The CCL6-CCR6 interaction regulates the migration of macrophages, neutrophils, astrocytes, and microglia in various inflammatory disorders^35^. Similar to CCL27, the IL-16-CD4 axis is related to increased sensitization, resulting in skin inflammation, and is involved in CD4+ T cell expansion^36^.

In contrast, OGDR media showed high expression of chemokines such as Hsp60 and CCL22. Hsp60 is a chaperone protein secreted under cellular stress or during immune responses^37^. Hsp60 is involved in autoimmune responses in the CNS and reduces inflammation in inflammatory disorders^38^. Although the Hsp60-TLR9/TLR2 interaction was an appealing target for this study, previous studies have shown that Hsp60 is not a direct factor in T cells^37^.

CCL22 is an effective chemoattractant for Treg cells and is secreted by monocytes^39^, which is in agreement with the chemokine profiling array results shown in this study. Notably, a few studies have shown a significant reduction in CCL22 levels during the acute phase of ischemic stroke in the circulation and infarct core, resulting in a higher National Institute of Health Stroke Scale (NIHSS)^40^. Recently, Rapp et al. reported that CCL22-CCR4 is a crucial factor in regulating the suppression of T cell activity through intervention by Treg cells^41^. Taken together, these results suggest that activated microglia release chemokines for pro-inflammatory immune cell trafficking and anti-inflammatory immune cell chemoattractants to balance detrimental neuroinflammation. The above observation was made in OGD media, but the level significantly increased in OGDR media in this study. However, in the context where the reduction of CCL22 during the initial phase of IS exacerbates the damage, the significance of CCL22-CCR4 in inducing Treg cell migration to alleviate ischemic stroke progression cannot be overlooked. Here, CXCR4 and CCR4 were chosen as the two potential target receptors because CCR4 blockade further inhibited Treg cell migration into the OGD media. Therefore, manipulation of CCR4 was the main target of this study.

Consistent with previous reports, a notable reduction in infarct size 24 h post-tMCAO in animals injected with either UT^17,42^ or TD was observed. The TD group showed further infarct size reduction. This may be due to a potential increase in the mobility of Treg cells in response to microglia-derived factors. This was supported by the transwell migration assay, which showed a notable increase in TD cell count in the OGD media. This is consistent with previous studies reporting the importance of the CCL22-CCR4 interaction in Treg cell migration^29,39,41^, suggesting the therapeutic potential of Treg cells to reduce ischemic damage and promote recovery in the brain post-stroke. Taken together, the importance of the CCL22-CCR4 interaction in reducing stroke severity via Treg cell recruitment may be a potent candidate for cell therapy. Various studies have shown the positive effects of the adoptive transfer of Treg cells into stroke patients, where they found reduced infarct volume and a better prognosis post-stroke^19,43^. However, some studies have reported that Treg cells do not reduce infarct size, while others have observed thrombosis formation through the CD40 and ICAM-1 pathways during Treg cell recruitment, worsening the damage^44,45^.

Our results suggest a novel idea of overexpressing CCR4 to enhance the therapeutic effects of Treg cells in response to microglia-derived chemokines in the acute phase of the ischemic stroke animal model. Despite the controversial effects of Treg cells in ischemic stroke concerning infarct size and functional outcome, our study showed a notable reduction in infarct size in the Treg cell injection group and a mild improvement in functional evaluation. Relating to previous studies regarding CCL22-CCR4 interactions in Treg cell trafficking and their ameliorating effects, the therapeutic potential of CCR4-overexpressing Treg cells can be further studied during the acute and recovery phases of ischemic stroke. However, further studies are required to validate the CCL22-CCR4 interaction quantitatively, and a more efficient increase in CCR4 in Treg cells is required.

### Supplementary Information

Supplementary Figures.

## Data Availability

All data produced and analyzed in this study are available from the authors upon reasonable request.
