# Supplementary material for: Ischemic stroke outcome after promoting CD4+CD25+ Treg cell migration through CCR4 overexpression in a tMCAO animal model
Source: Sci Rep. 2024 May 3;14:10201. doi: 10.1038/s41598-024-60358-2 (PMC11068779; doi:10.1038/s41598-024-60358-2)
Supplement: Supplementary file 1 — Supplementary Figures. [file 41598_2024_60358_MOESM1_ESM.docx]

**Supplementary material**


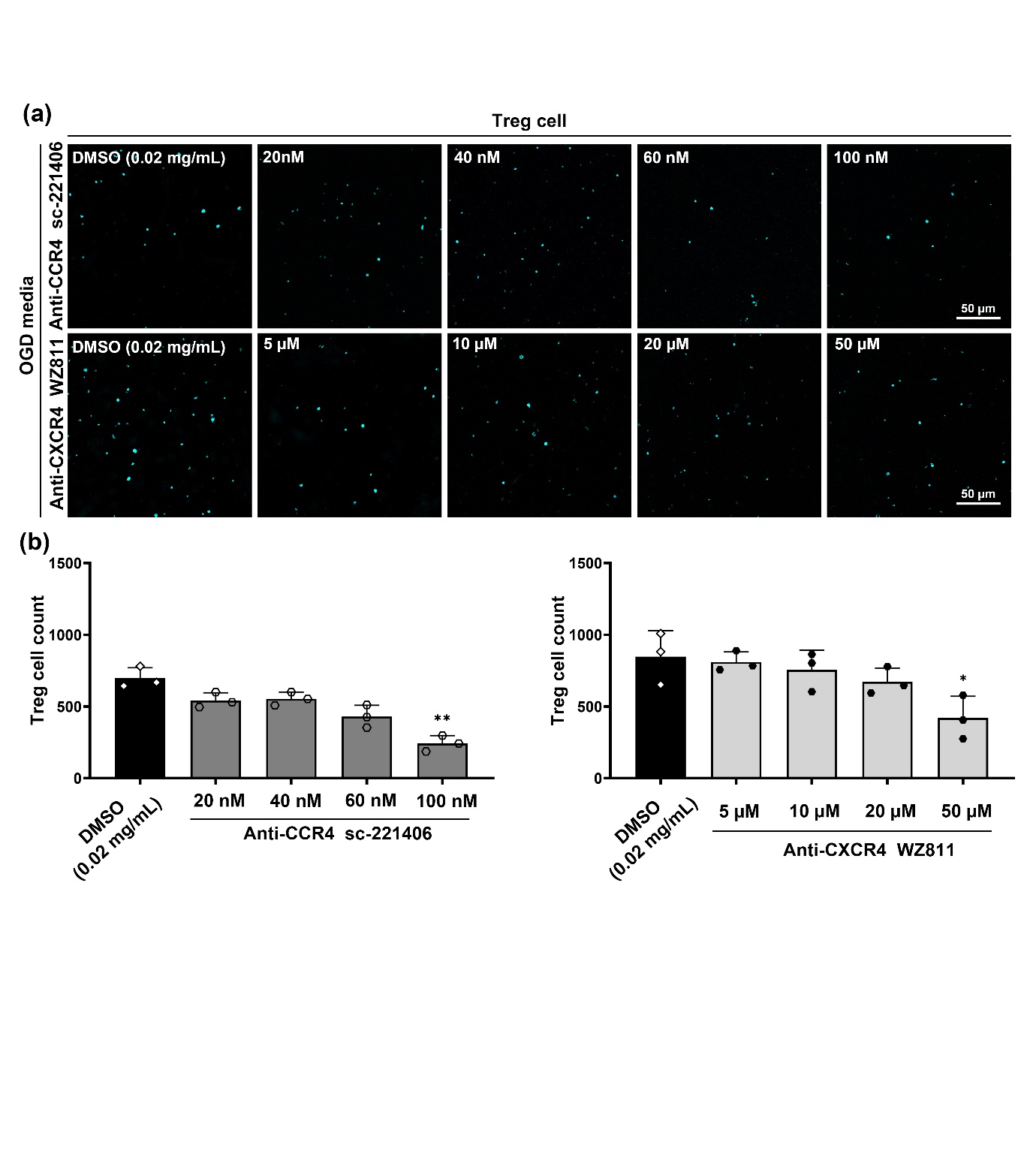


**Supplementary Fig. S1**. **Blocking CCR4 with the antagonist sc-221406 showed further inhibition of migration into the OGD media than blockage of CXCR4 with WZ811**. (**a**) Transwell migration assay showing CCR4 antagonist (sc-221406) or CXCR4 antagonist (WZ811) treated Treg cells (Scale bar = 50 µm). Each antagonist was treated at the indicated concentration for 2 h. (**b**) The Treg cell count data at each concentration indicated for each antagonist. Asterisks denote the significance of data from p-values obtained by performing the t-test (*p ≤ 0.05, **p ≤ 0.01, all n = 3).


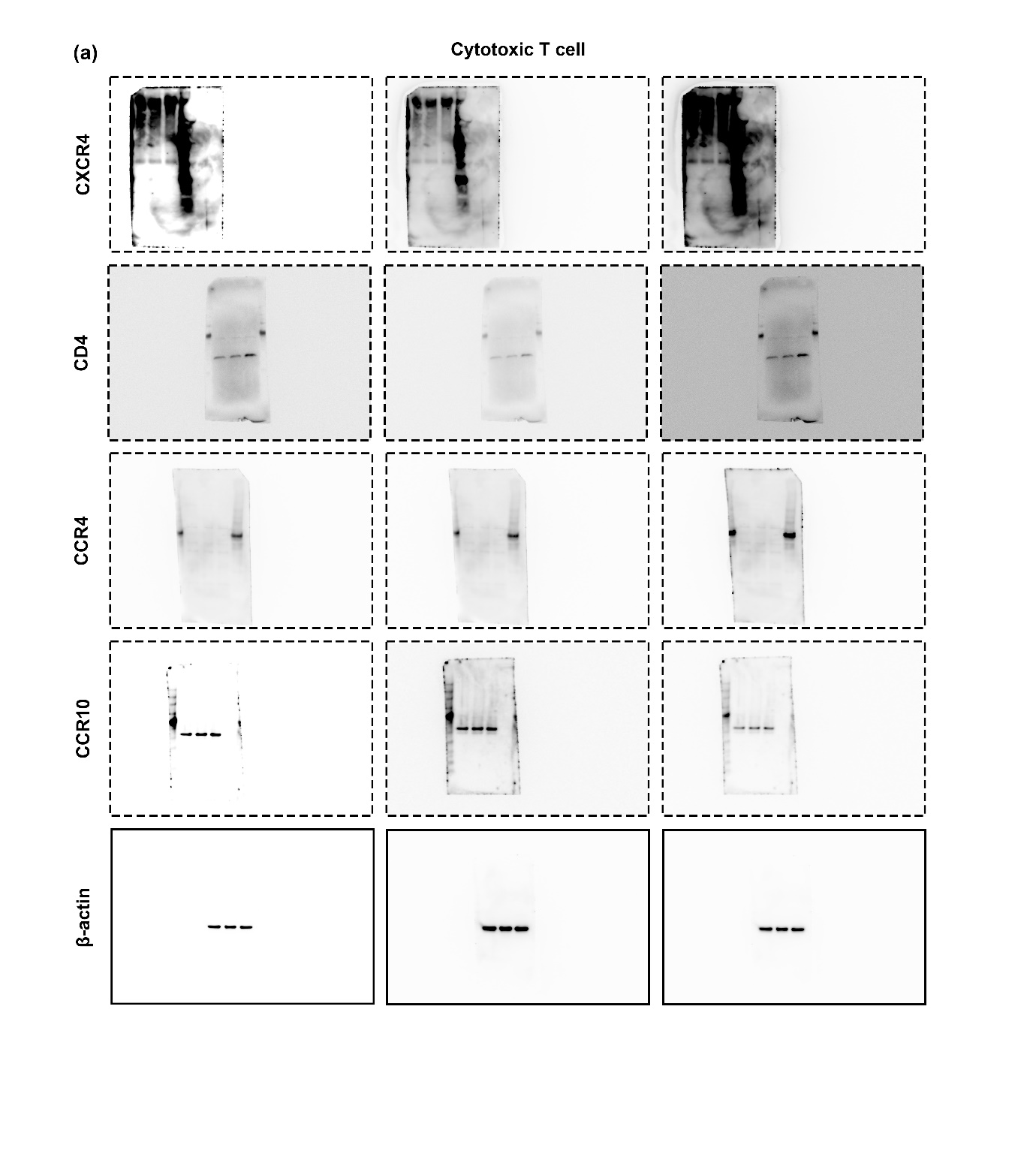

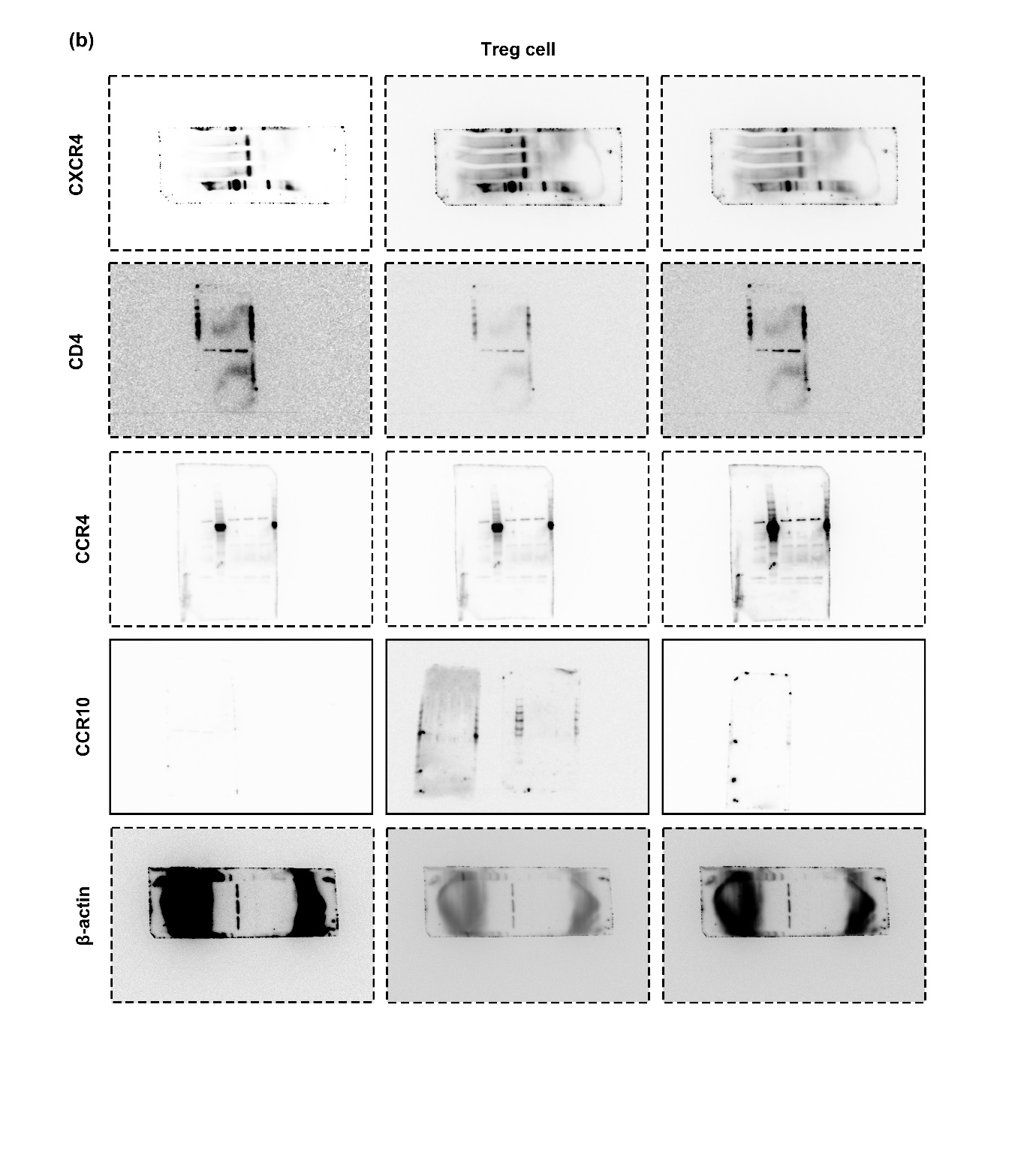


**Supplementary Fig. S2. Full length western blot membranes detecting each receptor from cytotoxic T cells (a) and Treg cells (b).** All receptors from each cell type were ran on separate gels and were transferred onto different membranes. Each membrane was trimmed before antibody incubation since the trimmed wells were empty or included the protein ladder. Each membrane was imaged under 120~150 sec exposure time. The dotted boxes include the same gel with different exposure time. The solid boxes include the different gels but of the same protein triplicate runs.
